# The Intake of Antioxidant Capacity of Children Depends on Their Health Status

**DOI:** 10.3390/nu14193965

**Published:** 2022-09-24

**Authors:** Beatriz Navajas-Porras, Sergio Pérez-Burillo, Daniel Hinojosa-Nogueira, Konstantinos Douros, Silvia Pastoriza, José Ángel Rufián-Henares

**Affiliations:** 1 Departamento de Nutrición y Bromatología, Instituto de Nutrición y Tecnología de Alimentos, Centro de Investigación Biomédica, Universidad de Granada, 18071 Granada, Spain; 2 Pediatric Allergy and Respiratory Unit, 3rd Department of Pediatrics, “Attikon” University Hospital, National and Kapodistrian University of Athens, School of Medicine, 11527 Athens, Greece; 3 Instituto de Investigación Biosanitaria ibs.GRANADA, Universidad de Granada, 18071 Granada, Spain

**Keywords:** antioxidant capacity, in vitro digestion–fermentation, children, gut microbiota, obesity, celiac disease, protein allergy, antioxidant intake

## Abstract

The gastrointestinal digestion of food and further gut microbial activity render a myriad of different molecules that could be responsible for the biological activities that are classically assigned to their parent compounds. This has been previously shown for some phytochemicals whose antioxidant capacity was either increased or decreased after being metabolized by gut microbes. Whether a global antioxidant capacity that is extracted from food is determined by the gut microbial community structure is still not well described. In the present study, we in vitro digested and fermented 48 different foods that were submitted to different culinary treatments using the stools of lean children, obese children, celiac children and children with an allergy to cow’s milk proteins. Their antioxidant capacities were assessed with the DPPH and FRAP assays, and the percentage that each food contributed to their daily antioxidant intake as well as their antioxidant capacity by portion size was inferred. Overall, cereals, fruits and vegetables displayed a higher contribution to their daily antioxidant intake, while tubers, fish and meat exhibited a higher antioxidant capacity by serving size. The food that was fermented in the lean children’s and those children that were allergic to cow’s milk protein’s fecal material, showed a higher antioxidant capacity, which could imply that there is a larger role of the gut microbiota in this area.

## 1. Introduction

The gut microbiota is the community of living microorganisms that reside and coexist in the gut. In the instance of humans, it consists of trillions of microbial cells and hundreds of different species, making it one of the most densely populated communities. It carries out a whole range of biochemical and physiological functions that influence the metabolism of the host [1], and it is responsible for the fermentation of food components. As a result, the gut microbiota is able to generate different compounds that directly affect human health in relation to nutrition, the regulation of immunity and systemic inflammation [2,3]. Among these compounds, we find some antioxidants that are transformed by the gut microbiota. This transformation is often essential for their absorption and thus, it is critical for them to exert their biological activity [4,5,6]. The inter-individual variability and plasticity that occurs has hampered the endeavors to define what a ‘healthy’ microbiota is. Therefore, markers of microbiota stability such as richness, diversity and functionality are often used as indicators of gut health due to their inverse association with certain pathologies. [1].

The gut microbiota has been identified as an influencing factor in the development of obesity by increasing the host’s capacity for energy harvesting [7]. Some alterations in the gut microbial community structure have been associated to obese people, including a decrease in the *Bacteroides* levels while there is an increase of the number of *Firmicutes*. It has also been observed that the microbiota of obese individuals has a lower microbial biodiversity than that of lean individuals [8]. Dysbiosis is a risk factor for celiac disease. This inflammation of the small intestine is characterized by a continuous gluten intolerance that is manifested in individuals with a genetic predisposition for it [9]. Gut microbial dysbiosis and some specific bacteria have been associated with celiac disease, either by increasing the inflammatory response to gluten or by directly influencing the mucosal immune responses [1,10]. Last but not least, food allergies have been also associated with disruptions in the gut microbial community structure [11,12,13,14].

Therefore, the objective of this paper is to study how different foods can contribute to the daily antioxidant intake of children after they have been fermented with fecal material from different child populations, and whether gut microbial differences play an important role in this or not.

## 2. Materials and Methods

### 2.1. Chemicals

#### 2.1.1. In Vitro Digestion and Fermentation

The pancreatin (from Alpha Aesar, Lancester, UK) was from porcine pancreas. The sodium di-hydrogen phosphate, tryptone, pepsin, porcine bile acids, cysteine, resazurin, sodium sulphide and salivary alpha-amylase were from Sigma-Aldrich (Darmstadt, Germany). 

#### 2.1.2. Antioxidant Capacity

The reagents DPPH (2,2 diphenyl-1-1picrythydrazul hydrate 95%), Trolox ((±)-6-Hydroxy-2,5,7,8-tetramethylchromane-2-carboxylic acid), iron (III) chloride hexahydrate, TPTZ (2,4,6-Tri(2-pyridyl)-s-triazine) and hydrochloric acid were from Sigma Aldrich (Darmstadt, Germany).

### 2.2. Samples

A total of 48 samples belonging to different food groups have been studied: cereals (biscuits, biscuits whole grain, bread, bread whole grain, breakfast cereals, breakfast cereals whole grain, penne (pasta), penne whole grain, rice and rice whole grain), cocoa (dark chocolate and Nutella), fruits (apple, banana, grapes, olives, orange, peach and plum), legumes (kidney beans and lentils), nuts (nut mixture and peanuts), oils (olive oil and sunflower oil), tubers (potato and sweet potato), vegetables (cabbage, carrot, cauliflower, eggplant, lettuce, onion, pepper, spinach, tomato and zucchini), dairy products (butter, gouda, milk and yogurt), eggs, fish (cod fish and salmon) and meat (beef, chicken, lamb and pork). The food items were purchased from 3 different retail shops and were stored at −80 °C until the experimental processing was performed.

### 2.3. In Vitro Digestion–Fermentation

The samples were submitted to in vitro digestion and fermentation following our previous protocols [15,16]. For each sample, 5 g of food (in triplicate) were weighed. In vitro digestion was divided into three steps: oral, gastric and intestinal. First, 5 mL of simulated saliva with 150 U/mL salivary alpha-amylase were added to 5 g of sample and blended in a 50 mL tube, keeping them at 37 °C for 2 min. Subsequently, 10 mL of simulated gastric fluid containing gastric pepsin (4000 U/mL) was added to the mixture, the pH was lowered to 3 and kept at 37 °C for 2 h. In the last step of the digestion, simulated intestinal fluid (20 mL with 200 U/mL pancreatin and 20 mM bile salts) was added to the tube, and the pH was raised to 7 and it was maintained at 37 °C for two hours. The enzymatic activity was stopped by its immersion in iced water for 15 min. The tubes were then centrifuged, the supernatant (fraction potentially absorbed in the small intestine) was collected and the pellet (undigested fraction that would reach the colon) was used for in vitro fermentation.

Fecal samples from 4 different groups of children were used for in vitro fermentation: 5 lean donors, 5 celiac donors, 5 obese donors and 5 donors with an allergy to cow’s milk proteins. The inclusion criterion for all of them was an age comprised from 8 to 10 years. Children taking probiotics or antibiotics in the previous 3 months were removed from the study. For the lean and obese groups, a common exclusion criterion was the diagnosis of chronic gastrointestinal disorders or any other chronic disease or being on a special diet. The BMI of the celiac, lean and milk-allergic children was comprised between the 5th and 85th percentile for their gender, height and age. For the obese group, their BMI had to be above the 95th percentile for sex, weight and age. Each stool sample was collected in a hospital in Athens (Greece) by the pediatric department. The informed consent document was signed by their parents. That form included all of the information of the study as well as the exclusion and inclusion criteria. The study was approved at the University General Hospital (Athens) by the corresponding ethical committee. 

The fecal material was combined by a group of donors to consider inter-individual variability. In vitro fermentation was performed in oscillation at 37 °C for 20 h. For this procedure, 0.5 g of the solid residue that was obtained after the in vitro gastrointestinal digestion plus a 10% of the in vitro supernatant were used.

The fermentation medium included cysteine 312 mg/L, resazurin 0.1% v/v, peptone 14 g/L and hydrogen sulphide 312 mg/L. Seven-point five mL of this fermentation medium was added to the fermentation tube. The inoculum was made from fecal material from each of the groups of children. Each of them was mixed with a PBS (at 33% concentration). Two mL of inoculum were added to the fermentation tube (each food sample was fermented 4 times, once for each inoculum). Then, in order to reach anaerobic conditions, the nitrogen was bubbled, thereby leaving a transparent solution (contrary to the pink color obtained under the presence of oxygen). The microbial activity, after fermentation at 37 °C for 20 h, was finished by the immersion of the tubes for 15 min in ice; then, centrifugation was conducted to collect the supernatant (the fraction that could be absorbed in the large intestine), which was finally stored for further analysis at −80 °C. The in vitro digestion *and* in vitro fermentation included blanks carrying water instead of the sample.

### 2.4. Antioxidant Analyses

The fraction that was obtained from the in vitro digestion was used to study its antioxidant capacity, since it is potentially absorbable in the small intestine. The liquid fraction that was obtained after the in vitro fermentation was also studied, since it could be absorbed in the large intestine. Both of the fractions can be summed to constitute the total antioxidant capacity of the sample [17]. Two different assays were used to analyze the antioxidant capacity using a microplate reader (FLUOStar Omega, BMG Labtech, Ortenberg, Germany).

The TEAC_FRAP_ method was performed following the protocol of Benzie and Strain [18]. With this method, the ability of the samples to reduce iron is studied. Twenty μL of sample was added to a 96-well microplate and mixed with 280 µL of FRAP reagent (freshly prepared). This reagent consisted of 25 mL sodium acetate (0.3 mM, pH 3.6), 2.5 mL ferric chloride, and 2.5 mL TPTZ 40 mM. The assay was monitored at 37 °C for half an hour at 595 nm, and the calibration curve ranged from 0.01–0.4 mg Trolox/mL. All of the samples were assessed in duplicate.

The TEAC_DPPH_ method was performed according to the method of Brand-Williams et al. [19]. Twenty μL of sample were added to a 96-well plate and blended with 280 μL of DPPH daily solution (at a concentration of 74 mg DPPH salt per L of methanol). The assay was followed at 520 nm for one hour at 37 °C, and the calibration curve ranged from 0.01 to 0.4 mg Trolox/mL. All of the samples were assessed in duplicate.

### 2.5. Daily Antioxidant Intake Calculations and Mean Contribution to Daily Antioxidant per Serving Intake

The contribution of each group of foods to the daily intake of the antioxidant capacity within the diet of children was calculated in two different ways. The first was by using the daily food intake according to the EFSA [20] and our results of antioxidant capacity with the following equation:

Daily Antioxidant Intake = Food daily consumption (g/day) × Antioxidant capacity (μmol/g)

The second way was by calculating the antioxidant capacity that was released by each group of food in terms of the usual serving size for children, according to a previous work [21] and our results of antioxidant capacity.

### 2.6. Statistical Analyses

Statistically significant differences were calculated with the unpaired Kruskal–Wallis test at 95% confidence, thereby comparing the contribution to antioxidant capacity of each food group with the mean baseline of antioxidant activity that was provided. Thus, we showed whether a particular food group has a higher or lower contribution to the antioxidant capacity of the diet than the mean. The Statgraphics Plus 5.1 software was used to compute all of the statistical analyses.

## 3. Results

In this study, the antioxidant power of foods were assessed in the liquid supernatant which was released after the in vitro digestion- fermentation. Thus, the total antioxidant capacity of a particular food is the sum of both of the fractions. Each of the food items was in vitro digested once but they were fermented with feces from each of the children. Therefore, the antioxidant capacity that was obtained during digestion was the same for each of the children. From these results, the daily antioxidant intake per food group was calculated, grouping each food into its corresponding group, and taking into account the EFSA data [20]. In the same way, the daily intake was calculated, taking into account the regular serving size for children [21]. The foods were grouped as is described in Section 2.2, and the averages were calculated to perform the calculations per group and for the percentages of the daily intake.

### 3.1. Daily Antioxidant Intake with the FRAP Method

**Figure 1:**
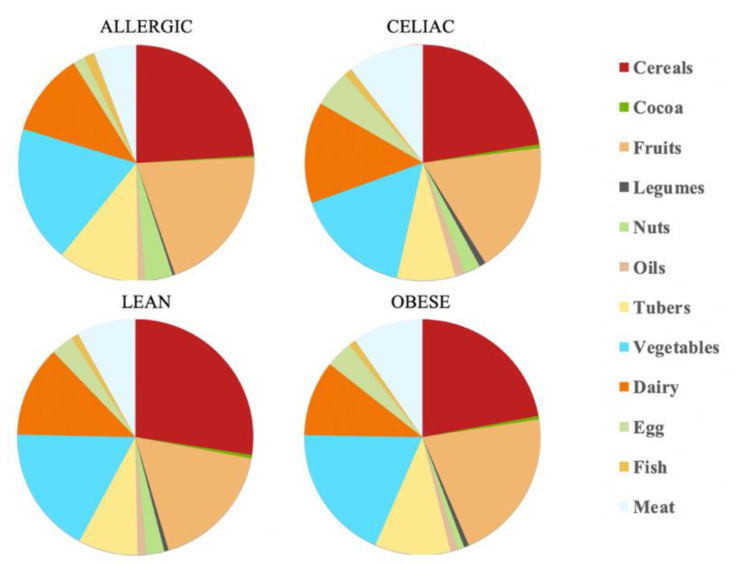
Mean contribution to daily antioxidant capacity intake (%) according to a FRAP assay in allergic, celiac, lean and obese children.

The total daily antioxidant intake was calculated for each child. According to EFSA [20] and our antioxidant capacity data, the daily antioxidant intake was: 111 mmol Trolox/day for the healthy lean children, 59.9 mmol Trolox/day for the obese children, 72.6 mmol Trolox/day for the celiac children and 142 mmol Trolox/day for the milk-allergy children. Therefore, the milk-allergy children were able to extract the highest antioxidant potential from their diet, whereas the obese children did the opposite. When we consider the specific food categories, cereals contributed the largest percentage of daily antioxidant intake among all the four groups of children: 27% for lean children, 22% for obese children, 22% for celiac children and 23% for allergic children. The fruits’ and vegetables’ contribution to daily antioxidant intake was only second to that of cereals. In contrast, cocoa and legumes showed the lowest contribution to daily antioxidant intake. Cocoa, specifically, contributed with 0.5% of it for the lean, obese and celiac children, and 0.2% of it for the allergic children (Table 1 and Figure 1).

**Figure 2:**
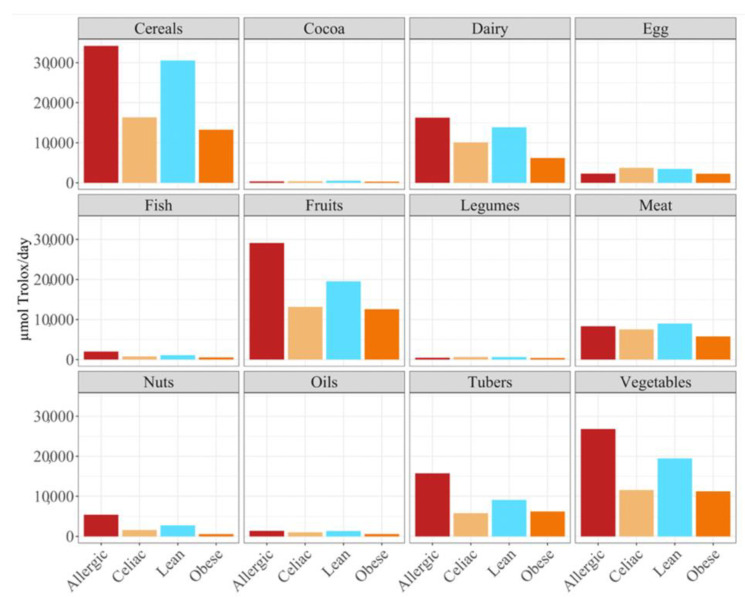
Differences in daily antioxidant intake in different foods groups in allergic, celiac, lean and obese children.

**Figure 3:**
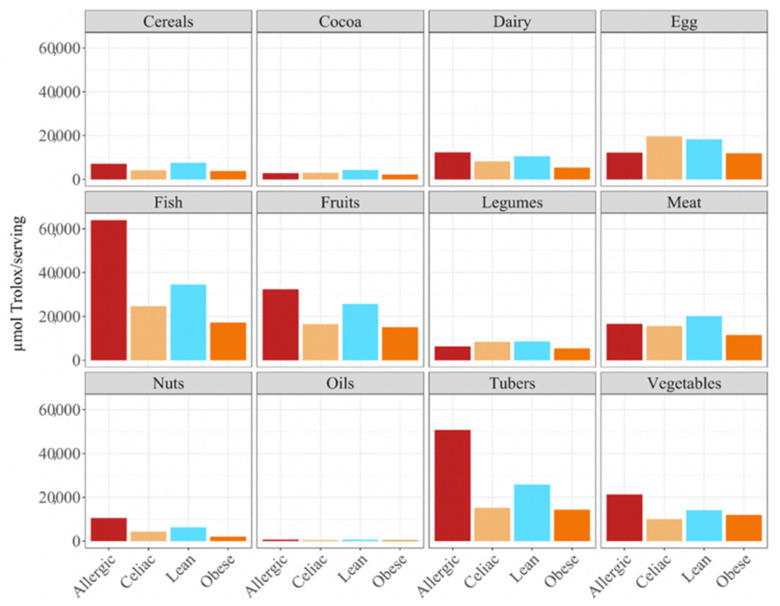
Differences in antioxidant intake per serving in different food groups in allergic, celiac, lean and obese children according to the FRAP method.

Overall, the food groups that showed the strongest contribution to daily antioxidant intake for all of the four population were cereals, dairy, fruits and vegetables (Figure 2). In fact, a statistically significant (*p* < 0.05) higher daily antioxidant intake was obtained for the children with a milk allergy and lean children in cereals and vegetables, while the daily intake of antioxidant capacity was only statistically higher for the children with a milk allergy in fruits, nuts and tubers. In terms of the daily antioxidant intake per serving size, fish was the highest contributing food group (28–44%), while oils contributed the least (0.5–0.7%) (Figure 3). The intake of the antioxidant capacity per serving was statistically higher (*p* < 0.05) for the children with a milk allergy in the case of fish, nuts and tubers. We also observed that the same food group always showed a lower contribution to daily antioxidant intake when such a food group was fermented using obese fecal material. No other tendencies such as this one were found though, as is showed by Figure 2, and the results were heavily influenced by the source of the fecal material that was used for fermentation. So, in conclusion, with the FRAP method it seems that children with a milk allergy are able to extract more antioxidant capacity from food and, on the contrary, obese children do the opposite.

When we studied the antioxidant capacity that each population was able to extract from a serving size, we observed that, as before, the antioxidant capacity was lower when the food was fermented with obese fecal material (Figure 3). Again, these results showed that different fecal materials meant that there would be differences in the antioxidant capacity that would be obtained by serving size. The highest antioxidant values were usually obtained when the foods were fermented using fecal material from children who were allergic to milk, although meat, eggs, legumes, cocoa, and cereals exhibited higher antioxidant values when they were fermented using healthy fecal material (Figure 3). 

### 3.2. Daily Antioxidant Intake with the DPPH Method

**Figure 4:**
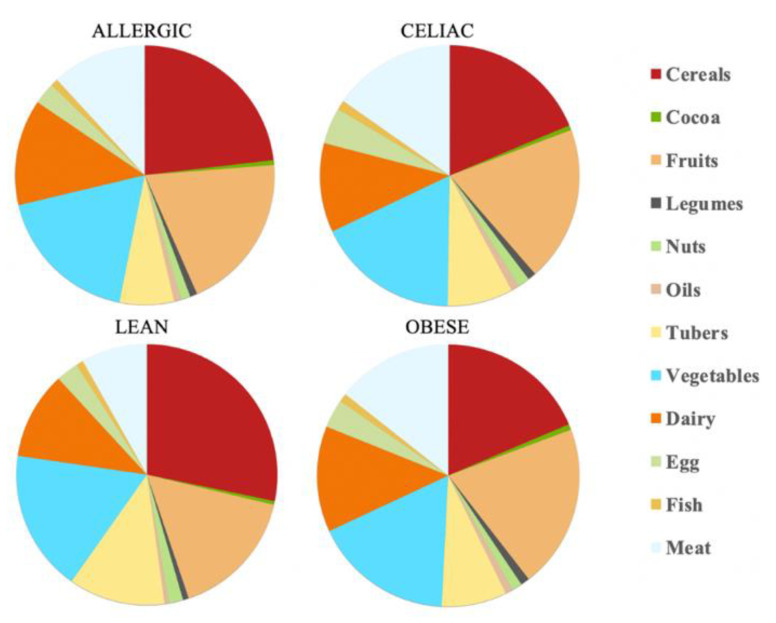
Mean contribution to daily antioxidant capacity intake (%) according to the DPPH method.

The calculations show that there is a daily antioxidant intake of 245 mmol Trolox/day for the healthy children and 80.3 mmol Trolox/day for the obese children, whereas, for the celiac children, this was 81.3 mmol Trolox/day and for the children with a milk allergy, this was 97.7 mmol Trolox/day. Therefore, the healthy children were the ones who were able to extract the higher antioxidant capacity values, at least daily. Again, the feal material source determined which food group was responsible for most of the daily antioxidant capacity. Thus, while the healthy children and those allergic to milk were able to scavenge more antioxidant capacity from cereals, the obese and the celiac children used fruits as their main antioxidant source. Regardless of the fecal material that was used for the fermentation, cereals, fruits and vegetables were always in the top three highest antioxidant capacity intake foods, which is in the same line as the results that were obtained by the FRAP assay. In contrast, cocoa and oil were the groups that had a lower contribution to the daily intake of antioxidant capacity (Table 2 and Figure 4).

**Figure 5:**
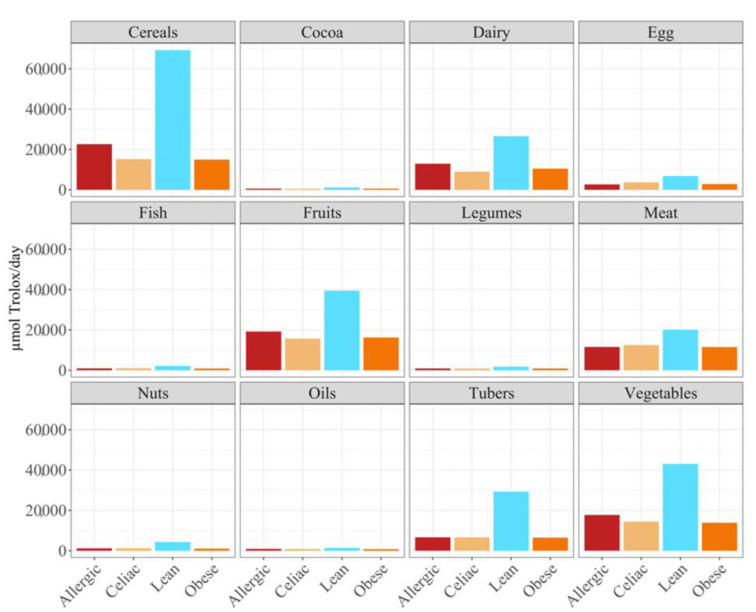
Differences in daily antioxidant intake in different food groups in allergic, celiac, lean and obese children.

As the FRAP assay showed before, the obese fecal material also extracted the lowest antioxidant values according to the DPPH assay, except for the dairy products and tubers (Figure 5). However, the main difference from the FRAP assay is that, here, the feces from the healthy, lean children were the one able to extract the highest antioxidant capacity (*p* < 0.05), either daily or by serving size (Figure 5), for the cereals, dairy, fruits, tubers and vegetables.

**Figure 6:**
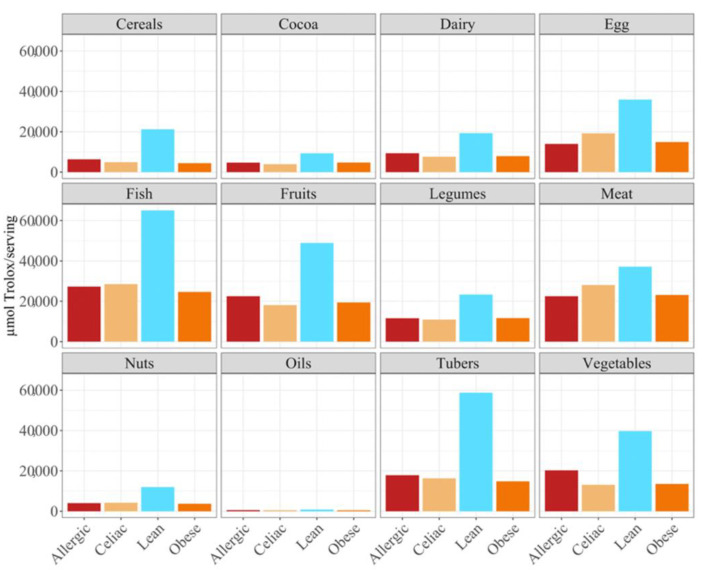
Differences in antioxidant serving intake in different food groups in allergic, celiac, lean and obese children according to the DPPH methods.

In terms of the contribution to daily antioxidant intake per serving, in all four groups of children, fish was the highest contributing food group (25–38%), while oils contributed the least (0.3–0.6%), as was true in the FRAP assay (Figure 6). Regarding the differences between the groups of children, the group of lean, healthy children had the highest antioxidant capacity per serving in all food groups, being statistically significant (*p* < 0.05) for cereals, dairy, fish, fruits, legumes nuts, tubers and vegetables. This was followed by the group of children with a milk allergy, except for fish, meat and nuts, where the second place was occupied by the celiac children. The group of obese children ranked last in almost all of the food groups, except for fruits, cocoa, tubers and vegetables, where the celiac children had the lowest antioxidant capacity per serving. (Figure 6). 

## 4. Discussion

At present, there is not much scientific literature on antioxidant capacity intake. Saura-Calixto and Goñi [22] studied it for an adult population, and did not take into account the physiological processes that food undergoes, such as digestion and fermentation. These processes considerably increase the antioxidants that are ingested by degrading the compounds and releasing others with a higher antioxidant capacity [15,22]. Furthermore, they only studied foods of plant origin, when in fact, foods with an animal source also provide a high quantity of compounds with an antioxidant capacity, such as dipeptides, polyamines, uric acid, B vitamins, among others [23]. In our previous work [24,25], foods of animal and vegetable origin were taken into account for the intake of compounds with an antioxidant activity in a Spanish diet for an adult population. In the results that were obtained for these adults, the foods that contributed most to the daily intake of antioxidant capacity were dairy products and meats, while for children, it was cereals and vegetables, except in the group of the obese children where fruit made a greater contribution than cereals did. Overall, cereals contributed between 22–27% for the FRAP method in all four groups of children and between 19–28% for the DPPH trial (except in the obese children, where fruit contributed 20% and cereals only 17%). Vegetables contributed between 16–19% for the FRAP method and between 17–18% for the DPPH method.

For the adults, the foods that contributed the most when using a serving size were fish and meat. For the children, these food groups were tubers and fish (30–45%) for the FRAP method, except in those children with celiac disease, where the foods that contributed the most antioxidant capacity per serving were fish and eggs (27%). For the DPPH method, the results coincided with those of the adults, with meat (23–34%) and fish (27–35%) being the food groups with the strongest antioxidant capacity per serving, except in the lean children, where, as in the FRAP study, fish and tubers (24%) were the groups of food with the strongest antioxidant capacity per serving.

Differences in the estimated daily food intake as well as in the serving size between the adult and child populations could be behind these disagreements regarding antioxidant capacity. Furthermore, while for the adult population Spanish reference intakes were used [26,27], for the children population we decided to use Greek references that were obtained for the children since the fecal material that was obtained was precisely from Greek children [20,21]. However, focusing on the serving size, both studies agree that the high antioxidant capacity that is found in meat and fish could be explained by those compounds that were mentioned earlier as well as by the feeding of the animals themselves [28]. 

The DPPH antioxidant values were usually higher than those which were obtained via the FRAP assay, for the lean children (245 mmol Trolox/day in DPPH > 111 mmol Trolox/day in FRAP), the obese children (80.3 mmol Trolox/day for DPPH vs. > 59.9 mmol Trolox/day for FRAP) and the celiac children (81.3 mmol Trolox/day for DPPH > 72.6 mmol Trolox/day for FRAP). However, the opposite was true for the children that had a milk allergy (97.6 mmol Trolox/day for DPPH < 142 mmol Trolox/day for FRAP). 

## 5. Conclusions

In the current paper, we studied the antioxidant intake per day and per serving in three groups of children with different pathologies, as well as in lean, healthy children. The differences between the groups suggest that the gut microbiota has a fundamental role in the release of compounds with an antioxidant capacity when fermentation takes place at the colonic level. However, since no further investigation of the gut microbial community structure was performed, via 16 rRNA or any other, we cannot ensure that our results are actually due to different microbial community structures. For the FRAP method, the group that was able to produce the best results (and therefore ingest more antioxidant compounds) were the children who were allergic to cow’s milk proteins, which could mean that their microbiota generates compounds with a greater capacity to reduce iron than the other groups. The lean children were in second place, and celiac and obese children were in last places, respectively. In the case of the DPPH, it was lean children who ingested the most amount of compounds with an antioxidant capacity, daily. Cereals, vegetables, and fruits stood out for their contribution to the daily antioxidant intake. On the other hand, tubers, fish, and meats stood out for their contribution to antioxidant intake per serving size. Few authors study daily antioxidant intake, and if they do, they do not consider the process of digestion and fermentation of foods during which many antioxidant compounds are generated through the metabolization of others. Foods with an animal source are also often neglected in such scientific studies. These conclusions highlight the need for further research in this area, as the scientific literature is scarce and incomplete.

## Figures and Tables

**Table 1:** Contribution of food consumption to the daily antioxidant capacity (AOX) intake in the children’s diet according to a FRAP assay.

Food Group	Child	AOX/Daily Intake (µmol Trolox/Day)	AOX/Serving Intake ^1^ (µmol Trolox/Serving)	Mean Contribution to Daily Antioxidant Intake (%)	Mean Contribution to Daily Antioxidant per Serving Intake (%)
Cereals	Lean	30,550	7620	27.4	6.84
Cocoa	Lean	537	4284	0.48	3.85
Fruits	Lean	19,544	25,697	17.5	23.1
Legumes	Lean	648	8600	0.58	7.72
Nuts	Lean	2739	6280	2.46	5.64
Oils	Lean	1328	709	1.19	0.64
Tubers	Lean	9105	25,891	8.17	23.2
Vegetables	Lean	19,487	14,145	17.5	12.7
Dairy	Lean	13,884	10,583	12.5	9.50
Egg	Lean	3487	18,334	3.13	16.5
Fish	Lean	1089	34,598	0.98	31.1
Meat	Lean	8999	20,143	8.08	18.1
Cereals	Obese	13,264	3799	22.1	6.33
Cocoa	Obese	283	2217	0.47	3.70
Fruits	Obese	12,576	15,136	21.0	25.2
Legumes	Obese	405	5363	0.67	8.94
Nuts	Obese	569	2029	0.95	3.38
Oils	Obese	592	408	0.99	0.68
Tubers	Obese	6210	14,305	10.4	23.9
Vegetables	Obese	11,243	12,004	18.7	20.0
Dairy	Obese	6212	5365	10.4	8.95
Egg	Obese	2260	11,883	3.77	19.8
Fish	Obese	566	17,219	0.94	28.7
Meat	Obese	5788	11,555	9.65	19.3
Cereals	Celiac	16,337	4159	22.5	5.72
Cocoa	Celiac	375	2980	0.52	4.10
Fruits	Celiac	13,179	16,514	18.1	22.7
Legumes	Celiac	633	8397	0.87	11.6
Nuts	Celiac	1606	4344	2.21	5.98
Oils	Celiac	996	531	1.37	0.73
Tubers	Celiac	5787	15,207	7.96	20.9
Vegetables	Celiac	11,566	10,063	15.9	13.8
Dairy	Celiac	10,105	8228	13.9	11.3
Egg	Celiac	3738	19,656	5.14	27.0
Fish	Celiac	792	24,705	1.09	34.0
Meat	Celiac	7559	15604	10.4	21.5
Cereals	Allergic	34,182	7153	24.0	5.02
Cocoa	Allergic	340	2813	0.24	1.98
Fruits	Allergic	29,123	32,369	20.5	22.7
Legumes	Allergic	475	6310	0.33	4.43
Nuts	Allergic	54,078	10,517	3.80	7.38
Oils	Allergic	1367	724	0.96	0.51
Tubers	Allergic	15,758	50,781	11.1	35.7
Vegetables	Allergic	26,811	21,372	18.8	15.0
Dairy	Allergic	16,272	12,268	11.4	8.61
Egg	Allergic	2316	12,176	1.63	8.55
Fish	Allergic	2028	63,938	1.42	44.9
Meat	Allergic	8332	16,568	5.85	11.6

^1^ Considering the intake of 1 serving.

**Table 2:** Contribution of food consumption to the daily antioxidant capacity (AOX) intake in the children’ diet according to the FRAP assay.

Food Group	Child	AOX/Daily Intake (µmol Trolox/Day)	AOX/Serving Intake ^1^ (µmol Trolox/Serving)	Mean Contribution to Daily Antioxidant Intake (%)	Mean Contribution to Daily Antioxidant per Serving Intake (%)
Cereals	Lean	69,285	21,222	28.3	8.66
Cocoa	Lean	1131	9290	0.46	3.79
Fruits	Lean	39,462	48,910	16.1	20.0
Legumes	Lean	1751	23,332	0.71	9.52
Nuts	Lean	4277	11,966	1.74	4.88
Oils	Lean	1376	844	0.56	0.34
Tubers	Lean	29,273	58,727	11.9	24.0
Vegetables	Lean	43,011	39,670	17.5	16.2
Dairy	Lean	26,546	19,352	10.8	7.89
Egg	Lean	6830	35,911	2.79	14.6
Fish	Lean	2161	65,047	0.88	26.5
Meat	Lean	8999	20,143	8.08	18.1
Cereals	Obese	14,937	4387	18.6	5.46
Cocoa	Obese	560	4750	0.70	5.92
Fruits	Obese	16,215	19,408	20.2	24.2
Legumes	Obese	882	11,714	1.10	14.6
Nuts	Obese	1001	3720	1.25	4.63
Oils	Obese	751	444	0.94	0.55
Tubers	Obese	6445	14,801	8.0	18.4
Vegetables	Obese	13,848	13,540	17.2	16.9
Dairy	Obese	10,473	7958	13.0	9.91
Egg	Obese	2839	14,929	3.54	18.6
Fish	Obese	828	24,599	1.03	30.6
Meat	Obese	11,521	23,155	14.35	28.8
Cereals	Celiac	15,222	4917	18.7	6.04
Cocoa	Celiac	490	3936	0.60	4.84
Fruits	Celiac	15,667	18,125	19.3	22.3
Legumes	Celiac	824	10,942	1.01	13.5
Nuts	Celiac	1196	4218	1.47	5.19
Oils	Celiac	832	487	1.02	0.60
Tubers	Celiac	6648	16,274	8.20	20.0
Vegetables	Celiac	14,410	13,117	17.7	16.1
Dairy	Celiac	8985	7675	11.0	9.44
Egg	Celiac	3659	19,242	4.50	23.7
Fish	Celiac	958	28,529	1.18	35.1
Meat	Celiac	12,456	28,102	15.31	34.5
Cereals	Allergic	22,607	6289	23.1	6.44
Cocoa	Allergic	586	4729	0.60	4.84
Fruits	Allergic	19,192	22,530	19.7	23.1
Legumes	Allergic	873	11,623	0.89	11.9
Nuts	Allergic	1147	4039	1.17	4.14
Oils	Allergic	864	527	0.89	0.54
Tubers	Allergic	6622	17,876	6.78	18.3
Vegetables	Allergic	17,708	20,284	18.1	20.8
Dairy	Allergic	12,923	9343	13.2	9.57
Egg	Allergic	2658	13,974	2.72	14.3
Fish	Allergic	910	27,299	0.93	28.0
Meat	Allergic	11,566	22,532	11.8	23.1

^1^ Considering the intake of 1 serving.

## Data Availability

Raw data are available upon request to Pro. Rufián-Henares.
